# Clostridium perfringens enterotoxin induces claudin-4 to activate YAP in oral squamous cell carcinomas

**DOI:** 10.18632/oncotarget.27424

**Published:** 2020-01-28

**Authors:** Chie Nakashima, Kazuhiko Yamamoto, Shingo Kishi, Takamitsu Sasaki, Hitoshi Ohmori, Rina Fujiwara-Tani, Shiori Mori, Isao Kawahara, Yukiko Nishiguchi, Takuya Mori, Masuo Kondoh, Yi Luo, Tadaaki Kirita, Hiroki Kuniyasu

**Affiliations:** ^1^ Department of Molecular Pathology, Nara Medical University, Kashihara, Nara 634-8521, Japan; ^2^ Department of Oral and Maxillofacial Surgery, Nara Medical University, Kashihara, Nara 634-8522, Japan; ^3^ Drug Innovation Center, Graduate School of Pharmaceutical Sciences, Osaka University, Suita, Osaka 565-0871, Japan; ^4^ Key Laboratory of Neuroregeneration of Jiangsu and Ministry of Education, Co-Innovation Center of Neuroregeneration, Nantong University, Nantong, Jiangsu Province 226001, China

**Keywords:** clostridium perfringens enterotoxin, claudin-4, YAP, Hippo signal, oral squamous cell carcinoma, Pathology

## Abstract

Claudin (CLDN)-4 expression has been associated with malignancy in various cancers. When CLDN4 expression was examined in oral squamous cell carcinoma (OSCC), 22 out of 57 (39%) cases showed immunoreactivity in the nucleus. Nuclear CLDN4-positive cases showed a stronger correlation with cancer progression than the negative cases. Intratumoral anaerobic bacterial DNA examination revealed nuclear CLDN4 expression in 81% of *Clostridium perfringens*-positive cases. Treatment of human oral squamous cell carcinoma cell lines HSC3 and HSC4 with *Clostridium perfringens* enterotoxin (CPE), induced CLDN4 nuclear translocation to enhance epithelial-mesenchymal transition (EMT), stemness, cell proliferation and invasive ability. In addition, CPE treatment suppressed phosphorylation of yes-associated protein-1 (YAP1) and promoted YAP1 nuclear translocation, resulting in increased expression of YAP1 target genes; cyclin D1 and connective tissue growth factor. Moreover, it was revealed that the complex of YAP1, CLDN4 and zona occludens-2 (ZO-2) was formed by CPE treatment, further suppressing YAP1 phosphorylation by LATS1 and activating it. Thus YAP activation in OSCC was regarded important in promoting malignant phenotypes. Our research suggested that the control of oral anaerobic bacteria may suppress YAP activation and in turn tumor progression.

## INTRODUCTION

More than 300 species of bacteria are found in the oral cavity, 10% of which are anaerobic in nature [1]. The number of bacteria in the oral cavity is determined by scraping the gingiva. Mostly the normal count of bacteria is about 10^11^ to 10^12^ cfu/ml and of these 0.1% are anaerobic [1]. Anaerobic bacteria in oral bacteria are regarded as the cause of periodontal diseases [2]. The number of anaerobes like *Peptostreptococcus*, *Prevotella*, and *Fusobacterium* are increased in the periodontal pocket of smokers and a strong correlation with periodontitis has been noted in such cases [3].

The anaerobic bacteria *Clostridium* is found in the plaque-associated bacterial flora in the oral cavity and is involved in the formation of dental caries [4]. It is also involved in the establishment of bacterial flora in the stomach and small intestine [1]. However, it is also known to damage the intestinal mucosa and cause enteritis by binding tightly to claudin (CLDN)-3 and CLDN4 and inhibiting intestinal tight junction barriers [5, 6].

According to many studies CLDN4 is overexpressed in many epithelial malignancies and it has been correlated with cancer progression [7–14]. CLDN4 expression is considered as a marker of epithelial differentiation [15–17], and its reduced expression is associated with epithelial-mesenchymal transition (EMT) [18, 19]. It forms tight junctions, maintains the cancer microenvironment, accumulates growth factors, and inhibits the penetration of anticancer drugs in the cells [12–14, 20]. In contrast, CLDN4 proteins which do not form tight junctions, act as ligand for integrin signaling and promote survival signals and stemness as found in undifferentiated gastric cancer cells [14].

Expression of CLDN4 is also affected by inflammatory cytokines. For instance, TNF-α suppresses the expression of CLDN4 in colorectal cancer, and the level of TNF-α is increased by *Clostridium perfringens* type A enterotoxin (CPE) [13]. In gastric cancer, the expression of CLDN4 is modulated by *Helicobacter pylori* [14, 21]. Thus, expression of CLDN4 is thought to be affected by the presence of bacteria within the tumor environment.

The oral microbiome population might promote carcinogenesis by increasing oxidative stress in the oral cavity of people with adverse lifestyle habits such as smoking, alcohol drinking and betel chewing [22]. But it is still unclear what role the oral bacteria play exactly in the development of oral cancer.

CLDN along with occludin are important components of tight junctions. Tight junctions activate Hippo signaling through cell adhesion and suppress proliferation of cells [23, 24]. In contrast, zonula occludens (ZO)-1 and ZO-2, which are lining proteins in tight junctions, activate yes-associated protein (YAP) and transcriptional coactivator with PDZ-binding motif (TAZ) by inhibiting the Hippo signaling pathway [25, 26], promoting cancer cell growth and epithelial-mesenchymal transition (EMT) to enhance cancer metastasis [27–29].

In this study, we examined the role of the bacteria in the oral cavity, especially anaerobic bacteria, in the development of oral cancer through the action on CLDN protein. We showed that anaerobic bacteria impair tight junctions and promote cancer progression through YAP1 activation in oral squamous cell carcinomas (OSCCs).

## RESULTS

### Expression of CLDN4 in OSCCs

**Figure 1:**
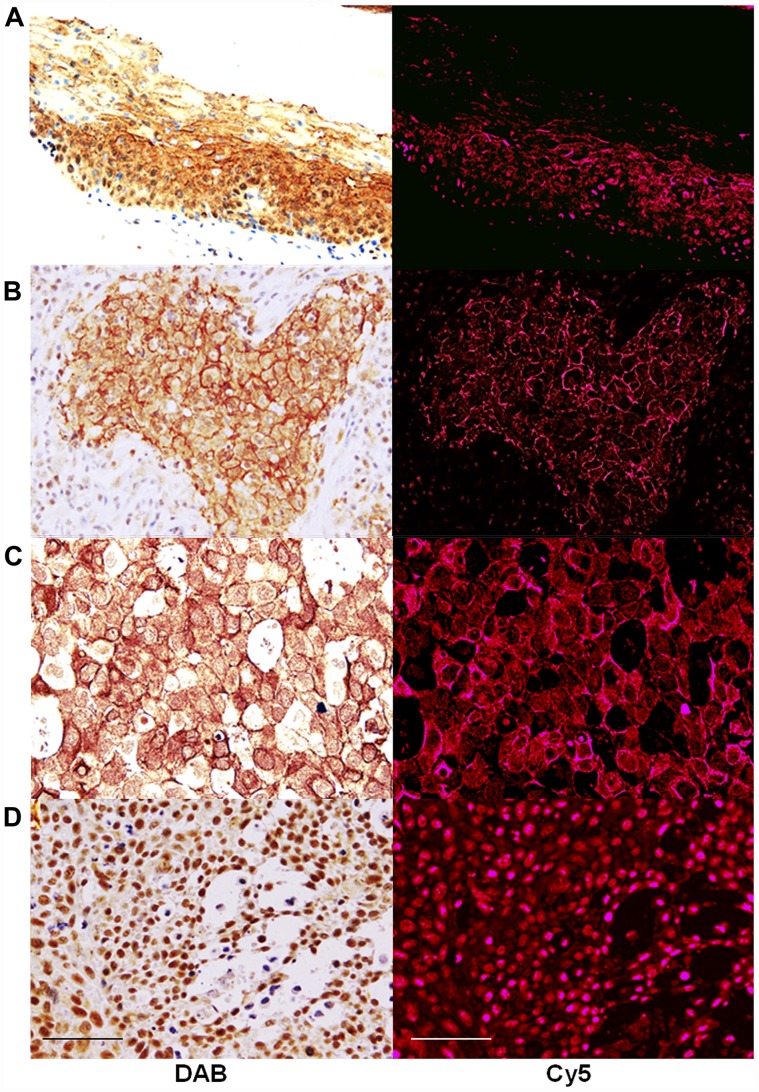
Expression of CLDN4 in oral squamous cell carcinomas. Immunohistochemical evaluation was carried out to identify CLDN4 using anti-CLDN4 antibody, 4D3. CLDN4 was visualized by peroxidase-diaminobenzidine (DAB) method (left column) or Cy5-labeled secondary antibody (right column). Cy5 images were semi-quantified for evaluation of CLDN4 protein levels. (**A**) non-cancerous tongue epithelium (**B**) OSCC, G2, pT2pN0pM0, (**C**) OSCC, G2, pT3pN1pM0, (**D**) OSCC, G3, pT4PN2pM0. Bar, 100 μm.

Immunostaining was performed to examine the expression of CLDN4 in the samples collected from 57 cases of OSCCs (Figure 1). In the squamous epithelium of the non-cancerous oral mucosa, the staining of the nucleus was observed in the basal region and the staining of the cell membrane was observed in the surface layer (Figure 1A). On the other hand, OSCCs showed CLDN4 immunoreactivity in the cytoplasmic membrane (Figure 1B), the cytoplasm (Figure 1C), and the nuclei (Figure 1D). In cases where clear expression was observed in the cell membrane, nuclear CLDN4 expression was not observed.

CLDN4 protein expression was semi-quantitatively determined by fluorescence intensity (Figure 1 and Table 1). No correlation was found between the differentiation grade, pathological stage, tumor progression and CLDN4 expression. In contrast, there was a correlation between lymph node metastasis and CLDN4 expression in the nodal metastasis foci, which was higher than in the primary lesion.

**Table 1:** Expression of CLDN4 in oral squamous cell carcinomas

**Tumor stages/Histological grades**	***n***	**CLDN4 intensity**	***P***
Primary	57	72 ± 10	0.006
Lymph node	8	161 ± 39	
G1	10	54 ± 23	NS
G2	32	84 ± 16	
G3	15	60 ± 14	
pStage 1–2	34	50 ± 12	NS
pStage 3–4	23	89 ± 25	
pT1-2	25	53 ± 12	NS
pT3-4	32	87 ± 16	
pN0	40	68 ± 13	0.0098
pN1-2	17	81 ± 19	

Tumor stages and histological grades were determined according to the guidelines of Union for International Cancer Control TNM classification system [60].

Abbreviations: NS, not significant.

### Nuclear CLDN4 expression in OSCC cells

As observed in Figure 1, CLDN4 protein was frequently observed in the nuclei of OSCC cases. Cancer progression was compared between the cases in which nuclear CLDN4 was observed (including cases with positive expression in the nucleus and cytoplasm) and the cases in which CLDN4 expression was found in the cell membrane (Table 2). We observed that the progression, including tumor invasion and nodal metastasis, was more pronounced in nuclear CLDN4-positive cases than in cases where CLDN4 were observed in the cell membranes. Thus, CLDN4 expression in the nucleus was found to be associated with the progression of OSCC.

**Table 2:** Relationship between nuclear and membrane CLDN4 expression and clinicopathological parameters

**Tumor stage/Histopathological grade**	***n***	**CLDN4 expression**		***P***
		**Nuclear**	**Membrane**	
*n*		22	35	
G1	6	6	4	NS
G2	9	9	23	
G3	7	7	8	
pStage 1–2	14	7	22	0.0307
pStage 3–4	8	15	13	
pT1-2	19	3	21	0.0008
pT3-4	3	19	14	
pN0	13	9	25	0.029
pN1-2	9	13	10	

Tumor stages and histological grades were determined according to the guidelines of Union for International Cancer Control TNM classification system [60].

Abbreviations: NS, not significant.

### Production of nuclear CLDN4 by CPE

**Figure 2:**
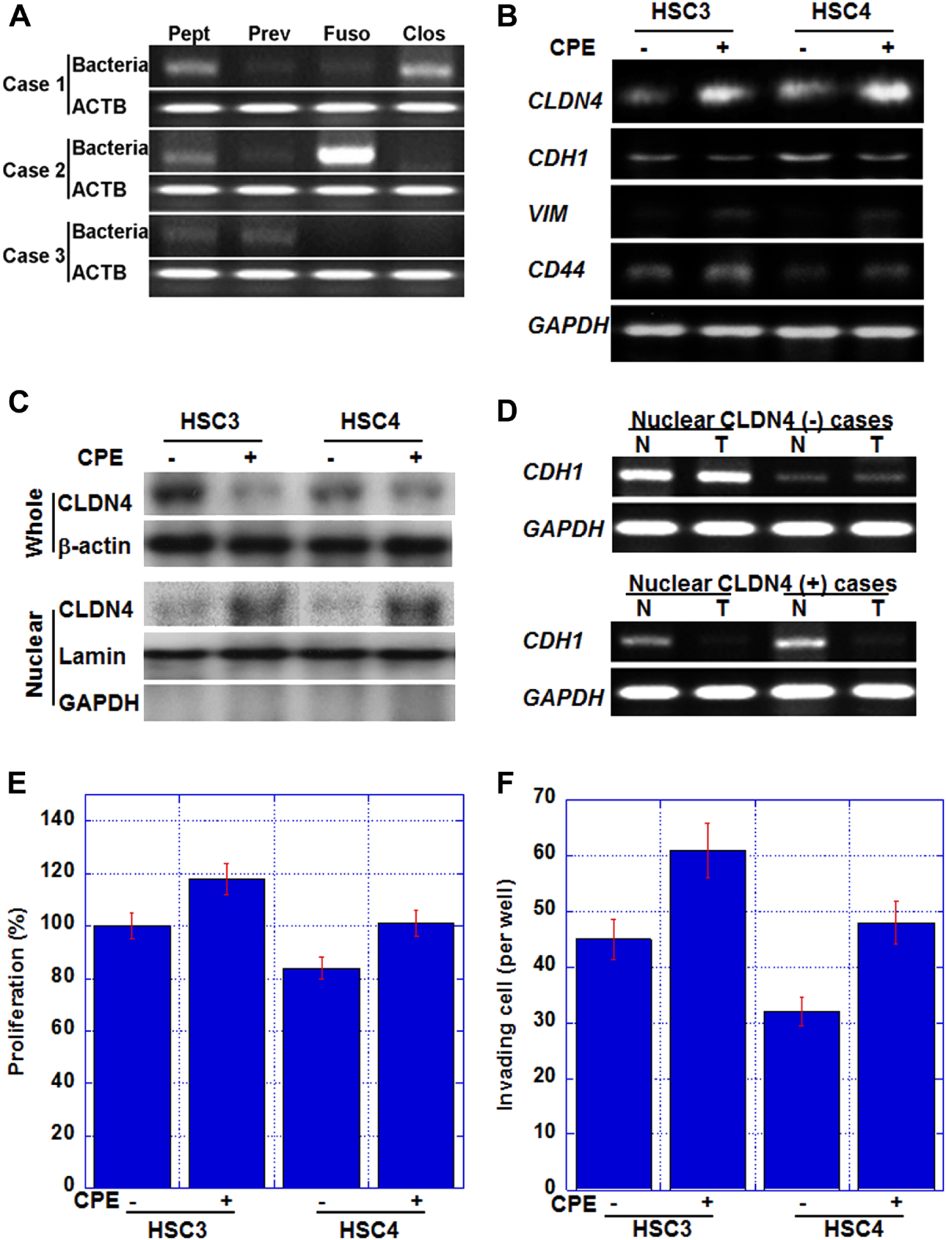
Effect of *Clostridium perfringens* enterotoxin (CPE) in human OSCC cell lines. (**A**) Intratumoral bacterial DNAs were detected by PCR. *Peptostreptcoccus* (Pept); *Prevotella* (Prev); *Fusobacterium* (Fuso); *C. perfringens* (Clos); ACTB, β-actin. (**B**) Gene expression in HSC3 and HSC4 human OSCC cells treated with CPE (10 μg/ml). CDH1, E-cadherin; VIM, vimentin; GAPDH, glyceraldehyde-3-phosphate dehydrogenase. GAPDH, loading control. (**C**) Nuclear translocation of CLDN4 in CPE-treated OSCC cells. Whole, whole cell lysate; Nuclear, nuclear fraction. β-actin, loading control for whole cell lysate; lamin, loading control for nuclear protein; GAPDH, a marker for cytoplasmic protein. (**D**) CDH1 expression in nuclear CLDN4-positive and negative OSCCs. T, tumor tissue; N, non-neoplastic epithelium; CDH1, E-cadherin; GAPDH, glyceraldehyde-3-phosphate dehydrogenase. GAPDH, loading control. (**E**, **F**) Effect of CPE on cell proliferation (E) and *in vitro* invasion (F). Bar, S. D. from 3 independent trials.

In nuclear CLDN4-positive cases, CLDN4 was not observed in the cell membrane (Figure 1), suggesting the possibility of some damage to the tight junction CLDN4. Since *Clostridium perfringens* enterotoxin (CPE) is a well-known factor that impairs CLDN4 [38], bacterial genome-specific sequences were amplified by PCR and examined for the presence of anaerobic bacteria in tumor tissues (Figure 2A). The presence of *Peptostreptococcus*, *Prevotella*, *Fusobacterium*, and *Clostridium* was examined in the tumor cells, and these anaerobic bacteria were found to thrive in the tumor cells in varying numbers. We examined the association of nuclear CLDN4 with *Clostridium*-negative and *Clostridium*-positive tumors. In 22 *Clostridium*-positive tumors, 18 (82%) showed nuclear CLDN4, whereas *Clostridium*-negative tumors showed no nuclear CLDN4 (*P* < 0.0001).

Next, we examined whether CPE was involved in the production of nuclear CLDN4 using human OSCC cells (Figure 2B and 2C). Our analysis revealed that CLDN4 mRNA levels and protein levels were increased in the nuclear fraction by CPE treatment in both the cell lines (Figure 2C); however, CLDN4 levels in whole cell lysate were decreased. These findings suggested that nuclear CLDN4 was produced by CPE.

The effects of nuclear CLDN4 on cancer cells by CPE treatment were also examined (Figure 2B). CPE treatment decreased the expression of E-cadherin in both the cell lines, while the mRNA expression of vimentin and CD44 increased suggesting EMT. We also examined E-cadherin mRNA expression in 2 nuclear CLDN4-positive and 2 CLDN4-negative OSCCs in Figure 2D. Nuclear CLDN4-positive tumors showed decreased E-cadherin mRNA expression in comparison with the non-neoplastic epithelium, whereas Nuclear CLDN4-negative tumors showed retained E-cadherin expression. Moreover, cell proliferation and invasive ability were enhanced by CPE (Figure 2E and 2F).

### Inhibition of Hippo suppression system by CPE

**Figure 3:**
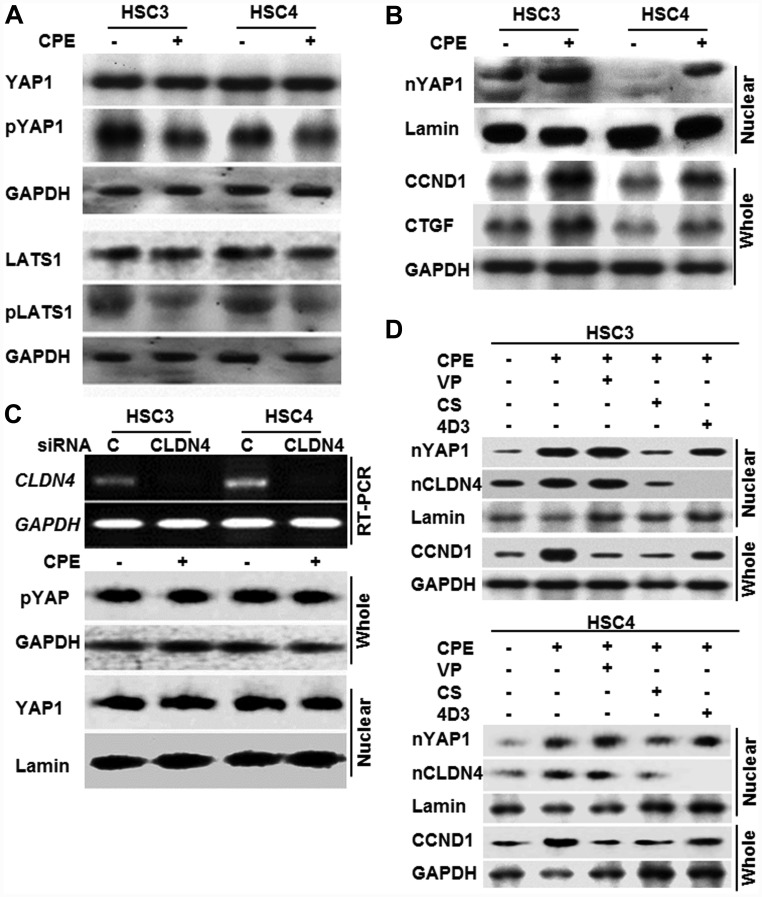
Effect of CPE on YAP1 activation in OSCC cells. (**A**) Phosphorylation of YAP1 and LATS1 in whole cell lysates of CPE (10 μg/ml)-treated OSCC cells. (**B**) Effect of CPE on expression of nuclear YAP1 (nYAP) and cyclin D1 (CCND1) and connecting tissue growth factor (CTGF). (**C**) Effect of CLDN4 knockdown on YAP1 activation. pYAP, phosphorylated YAP1. (**D**) Effect of YAP1 inhibitors and anti-CLDN4 antibody (4D3). VP, verteporfin; CS, cytostatin. Whole, whole cell lysate; Nuclear, nuclear fraction; lamin, loading control for nuclear protein; GAPDH, loading control for whole cell lysate.

In contrary of CLDN4 mRNA, CPE decreased the expression of CLDN4 protein and E-cadherin mRNA, suggesting decrease of cell adhesion. Since Hippo signaling pathway is activated by cell adhesion and suppresses YAP, the effect of CPE on the Hippo-YAP system was examined (Figure 3).

In both HSC3 and HSC4 cell lines, the levels of phosphorylated YAP1 and phosphorylated LATS were reduced by CPE treatment (Figure 3A). Along with this the nuclear YAP1 increased, in turn inducing its target genes, cyclin D1 (CCND1) and connective tissue growth factor (CTGF) (Figure 3B). In contrast, when CLDN4 was knocked down, its mRNA almost disappeared, and CPE-induced alterations in phosphorylated YAP1 and nuclear YAP1 were also abrogated (Figure 3C).

Next, the effect of the YAP inhibitors and anti-CLDN4 antibody (4D3) on the activation of YAP1 by CPE was examined (Figure 3D). CPE increased the nuclear YAP1 and CLDN4 and induced the YAP1 target gene CCND1. When treated with verteporfin, which inhibits YAP1-TEAD interaction [39], nuclear translocation of YAP1 and CLDN4 was maintained; however, CCND1 expression was suppressed. In contrast, treatment with cytostatin, a dephosphorylation inhibitor of phosphorylated YAP1 [40], suppressed both nuclear translocation of YAP1 and CLDN4 and decreased CCND1 expression. In contrast, when treated with the 4D3 antibody, CLDN4 nuclear translocation disappeared, and YAP1 nuclear translocation and CCND1 expression decreased.

### Interaction between YAP1 and CLDN4

**Figure 4:**
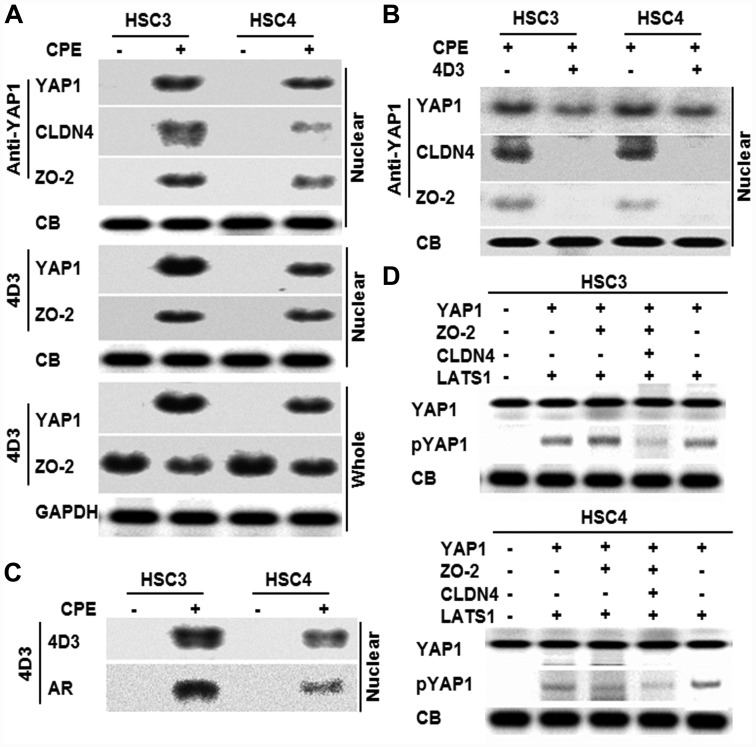
Effect of CPE on interaction of CLDN4 and YAP1 in OSCC cells. (**A**) Effect of CPE (10 μg/ml) on protein interaction among CLDN4, YAP1 and ZO-2 was examined by immunoprecipitation. (**B**) Nuclear translocation of CLDN4, YAP1 and ZO-2 in CPE-treated OSCC cells with or without 4D3 treatment. (**C**) CLDN4 nuclear translocation in the ^131^I-surface labeled OSCC cells. Nuclear fraction was immunoprecipitated with 4D3 for detection with 4D3 and autoradiography (AR). (**D**) *In vitro* phosphorylation assay using recombinant human YAP1, ZO-2, CLDN4 and LATS1. Reaction solution was subjected to immunoblotting. Whole, whole cell lysate; Nuclear, nuclear fraction; CB, Coomassie blue; lamin, loading control for nuclear protein; GAPDH, loading control for whole cell lysate.

Since interaction between YAP1 and CLDN4 was suggested above, immunoprecipitation was performed for these proteins and ZO-2, which had been reported to interact with YAP1 [25] (Figure 4A). When immunoprecipitation was performed with anti-YAP1 antibody in the nuclear fraction to detect YAP1, CLDN4, and ZO-2, their levels were found to be increased following CPE treatment. When the same nuclear fraction was immunoprecipitated with 4D3 antibody to detect YAP1 and ZO-2, the protein levels were found to be increased following CPE treatment. Furthermore, when the whole cell lysates were immunoprecipitated with 4D3 antibody to detect YAP1 and ZO-2, ZO-2 was also detected in CPE-untreated and CPE-treated cells; however, YAP1 was detected in only CPE treated cells. When nuclear fractions of cells treated with 4D3 antibody were precipitated with anti-YAP1 antibody to detect YAP1, CLDN4, and ZO-2 in CPE-treated OSCC cells, CLDN4 and ZO-2 were not detected. YAP1 was reduced by 4D3 treatment (Figure 4B). In order to examine whether CLDN4 on the cell membrane surface translocate to the nuclei, ^125^I was added to the culture medium simultaneously with the CPE treatment to label the protein on the cell membrane surface (Figure 4C). When the nuclear fraction was precipitated with the 4D3 antibody, an autoradiographic signal was detected in consistent with the signal detected with the 4D3 antibody. In order to examine the role of CLDN4 on YAP1 activation, *in vitro* phosphorylation of YAP1 was examined with or without CLDN4 (Figure 4D). YAP1 was phosphorylated by LATS1 with or without ZO-2, whereas mixture of YAP1, ZO-2 and CLDN4 showed reduction of YAP1 phosphorylation. In contrast, mixture of YAP1 and CLDN4 showed no reduction of YAP1 phosphorylation.

## DISCUSSION

In this study, OSCCs showing nuclear CLDN4 expression were found more strongly correlated with cancer progression than those showing cell membrane CLDN4 expression. Previous studies have shown that CLDN4 forms tight junctions that preserve the cancer microenvironment to accumulate growth factors and maintain extracellular pH in an acidic range [12–14, 20]. Tight junctions also inhibit the penetration of anticancer drugs into this microenvironment and develop resistance against anticancer drugs [12–14, 20]. Among the 57 cases of oral cancer examined in this study, intraoral CLDN4 was found in 22 (39%) of them. It was also observed that expression of cell membrane CLDN4 was never detected in nuclear CLDN4-positive cases. We assumed that this might be because cytoplasmic membrane CLDN4 damage was associated with CLDN4 nuclear translocation.

CPE is known as a factor that impedes tight junctions. Clostridia that produce CPE are part of the intestinal microflora and their numbers increase with age. However, *Clostridium* has also been reported to be associated with dental caries in the oral cavity [4]. Likewise, the presence of anaerobic bacteria such as *Peptostreptococcus*, *Prevotella*, and *Fusobacterium* in oral flora exacerbate periodontal disease [2, 3, 41].

Since the detection of *Clostridium* by culture methods is insufficient [42], we employed PCR-amplification of bacterial DNA to determine the frequency of *Clostridium* in oral flora. In general, *Clostridium* spp. are detected in the bronchial lavage of pneumonia patients by 16S ribosomal DNA-based bacterial flora analysis [43–46]. From these literature [43–46], we decided to detect four types of bacteria including *Clostridium* as anaerobic bacteria that are commonly found in the oral cavity using a similar approach. In the present study, tumor tissue was examined because the patient’s saliva could not be available. In the future, it is desired to clarify the relationship between bacterial flora and tumor progression through studies using saliva, which is easy to obtain without any pain for patients.

CLDN4 is bound to ZO-1 and ZO-2 in tight junctions [47]. Since CPE implicates homotypic binding of CLDN4 [38], it is thought to internalize in the cytoplasm through binding to ZO-1 or ZO-2. In addition, ZO-2 has binding properties with YAP1 through PDZ domain, and promotes nuclear translocation of YAP1. Our results suggested that the CLDN4-ZO-2 complex, which was internalized in the cytoplasm by CPE, further promoted YAP1 nuclear translocation by binding to YAP1. It was shown that YAP1 is less susceptible to phosphorylation by LATS1 when present in YAP1-ZO-2-CLDN4 or YAP1-ZO-2 complex. It is possible that the addition of CLDN4 to the YAP1-ZO-2 complex masks the phosphorylation site of YAP1.

Our data showed that YAP1-ZO-2-CLDN4 complex might activate YAP1. Tight junction-Hippo system contains molecular redundancy; CLDN3 instead of CLDN4, TAZ instead of YAP1, and ZO-1 instead of ZO-2 might form a complex protein reacting to CPE. For this reason, TAZ, ZO-1, and CLDN3 were examined in the nuclear fraction of OSCC cells treated with CPE as in Figure 3A, but were not detected (Supplementary Figure 1A). In addition, TAZ, ZO-1 and CLDN3 were examined by immunoprecipitation with the same anti-YAP1 antibody as in Figure 4A, but were not detected (Supplementary Figure 1B). In addition, TAZ was not detected in the precipitate from 4D3 antibody (Supplementary Figure 1B). These results suggested that a complex of YAP1-ZO-2-CLDN4 was formed preferentially for CPE. This specificity needs further study.

It has been reported that while the combination of YAP1-ZO-2 promotes cell death [46], the combination of YAP2-ZO-2 enhances tumor promoting activity [47] In our immunoprecipitation study, contrary to previous findings, ZO-2 and YAP1 formed a tumor-promoting complex and no ZO-2-YAP2 complex was observed (DNS). Regarding this difference, our results suggested that the formation of a complex of YAP1-ZO-2 and CLDN4 had some pro-tumoral effect. Tumor-promoting role of YAP is attributed to the induction of tumor-promoting gene expression via TEADS [40]. According to our findings, the expression of cyclin D1 and CTGF has a definite association with the formation of YAP1-ZO-2-CLDN4 complex.

Our results showed that *Cl. perfringens* might be a potent accelerator of malignant phenotype in OSCC. In a number of previous studies, *Clostridium* sp. have shown a strong relationship with cancer. For instance, *Cl. septicum* is known to be associated with colorectal cancer. It functions as an immunosuppressant and is frequently detected in the blood of colorectal cancer patients [48, 49]. The association between *Clostridium* in the blood and *Cl. perfringens,* and colorectal cancer risk has been reported as *P* = 0.13 and *P* = 0.17, respectively [48]. Inhibition of *Cl. leptum* subgroup by antibiotics has been reported to inhibit tumorigenesis in azoxymethane-dextran sulfate colon carcinogenesis model through reduction of aberrant DNA methylation [50]. In addition, colon carcinogenesis caused by a high-fat diet is associated with an increase in the intestinal bacterium *Clostridium* subcluster XIVa [51]. Another anaerobic bacteria, *Bacteroides fragilis*, promotes colon carcinogenesis by its enterotoxin through activation of Wnt signal by β-catenin nuclear translocation with E-cadherin cleavage and secretion of inflammatory cytokines [52]. Thus, anaerobic bacteria have shown a good association with colorectal cancer. However, there is no report of an association between anaerobic bacteria and cancer in tissues other than the colorectum. However, in our study, the activation of YAP1 by CPE promoted cell proliferation, invasion ability, stemness, EMT, and consequent cancer malignancy. Further it is needed to examine the association of *Cl. perfringens* with oral carcinogemesis.

CPE is regarded as a therapeutic tool for epithelial malignant tumors by utilizing the damage of CLDN4 and CLDN3 [11, 53, 54]. The 50%-inhibitory concentration of CPE for colon cancer cell lines was between 50-100 ng/ml [53]. The concentration of CPE used in this study was 10 ng/ml and no cytotoxicity was observed (DNS) at this concentration. Our data suggested that such low concentration of CPE was not cytotoxic to cancer cells and instead promoted malignant phenotypes.

YAP has been reported to be involved in the development and progression of lung cancer, liver cancer, and various other cancers [55–58]. However, recently, its involvement in squamous cell carcinoma and head and neck cancer has attracted attention [55]. Some studies have also shown an association between YAP and radiation resistance and poor prognosis [29, 59]. In head and neck cancer, YAP correlated with poor prognosis and resistance to treatment, suggesting a potential molecular target [29]. Our study inferred that *Clostridium* induced YAP1 activation, which in turn suggested that the suppression of *Clostridium* by antibiotics and/or oral hygiene might contribute to the suppression of carcinogenesis and cancer progression. Extensive clinical studies are required to prove this hypothesis in future.

## MATERIALS AND METHODS

### Surgical specimens

We reviewed the pathological diagnosis and clinical data of 57 patients diagnosed with OSCCs from 2004 to 2016, in the Department of Molecular Pathology, Nara Medical University, Japan. As written informed consents were not obtained from the patients, any identifying information was removed from the samples prior to analysis, in order to ensure strict privacy protection (unlinkable anonymization). All procedures were performed in accordance with the Ethical Guidelines for Human Genome/Gene Research issued by the Japanese Government and were approved by the Ethics Committee of Nara Medical University (approval number 937).

### Human OSCC cell lines

HSC-3 and HSC-4 human OSCC cell lines were purchased from Dainihon Pharmaceutical Co. (Tokyo, Japan). Cells were cultured in Dulbecco’s modified Eagle’s medium (DMEM) supplemented with 10% fetal bovine serum (FBS) at 37° C in 5% CO_2_. Cell growth was assessed using tetrazolium (MTT) dye assay, as previously described [30].

### Antibody and reagents

The anti-human CLDN4 extracellular domain antibody, 4D3, was developed by immunizing rats with a plasmid vector encoding human CLDN4 [12]. CPE was purchased from Sigma, USA (Sigma, St. Louis, MO, USA).

### Immunohistochemistry

Consecutive sections of 4 μm of OSCC were immunohistochemically stained using 0.2 μg/ml of 4D3 antibody by a previously described immunoperoxidase technique [31]. Secondary antibodies (peroxidase-conjugated and cy5-conjugated anti-mouse IgG rabbit antibodies, Medical and Biological Laboratories, Nagoya, Japan) were used at a concentration of 0.2 μg/ml. Tissue sections were color-developed with diamine benzidine hydrochloride (DAKO, Glastrup, Denmark) and counterstained with Meyer’s hematoxylin (Sigma, USA). Primary antibody was also detected by fluorescence microscopy (All-in-one fluorescence microscopy, BZ-X800, Keyence Japan, Osaka, Japan). Fluorescence intensity was measured according to the manufacturer’s instructions. The fluorescence intensity in the non-cancerous squamous cell epithelium was standardized and set to 100. For a negative control, non-immunized rat IgG (Santa-Cruz Biotechnology, Santa-Cruz, CA, USA) was used as the primary antibody.

### Immunoblot analysis

Whole-cell lysates of OSCC cells were prepared as previously described elsewhere [32]. Lysates (20 μg) were subjected to immunoblot analysis using sodium dodecyl sulfate polyacrylamide gel electrophoresis (SDS-PAGE, 12.5%), followed by electrotransfer onto nitrocellulose filters. The filters were incubated with primary antibodies, followed by peroxidase-conjugated IgG antibodies (Medical and Biological Laboratories). Anti-tubulin antibody was used to assess the levels of protein loaded per lane (Oncogene Research Products, Cambridge, MA, USA). The immune complex was visualized using an Enhanced Chemiluminescence Western-blot detection system (Amersham, Aylesbury, UK). Antibodies for CLDN4 (4D3), YAP1, phosphorylated YAP1 (pS127), zona occludens-2 (ZO-2), GAPDH (glyceraldehyde-3-phosphate dehydrogenase), cyclin D1 (CCND1), connective tissue growth factor (CTGF) (Abcam, Cambridge, UK), large tumor suppressor kinase 1 (LATS1), phosphorylated LATS (pThr1079) (Cell Signaling Technology, Beverly, MA, USA), β-actin, lamin (Zymed Laboratories Inc., South San Francisco, CA, USA), (Proteintech Group Inc., Rosemont, IL, USA) were used as primary antibodies.

### Bacterial DNA amplification

Bacterial DNA was extracted from OSCC specimen (10 thin-sliced paraffin-embedded tumor specimen, depraffinized, and hydrated) using the QIAamp DNA mini kit (Qiagen, GmbH, Hilden, Germany) according to the instructions of the manufacturer. The extracted DNA samples were stored at −20° C. PCR was carried out for 35 cycles and each cycle consisted of the following steps; denaturation (94° C for 5 min), annealing (50° C for 1 min) and primer extension (72° C for 1.5 min). Amplified PCR products were analyzed by 1.5% agar gel electrophoresis in Tris–Borate-EDTA buffer. The gel was stained with 0.5 μg/ml ethidium bromide. The primer sets used for the amplification of bacterial DNA for the following bacteria were as follows; *Peptostreptococcus productus*, forward, 5′-AAC TCC GGT GGT ATC AGA TG-3′ and reverse, 5′-GGG GCT TCT GAG TCA GGT A-3′ [33]; *Fusobacterium prausnitzii*, forward, 5′-AGA TGG CCT CGC GTC CGA-3′ and reverse, 5′-CCG AAG ACC TTC TTC CTC C-3′ [33]; *Prevotella nigrescens*, forward, 5′-GTG TTT CAT TGA CGG CAT CCG ATA TGA AAC-3′ and reverse, 5′-CCA CGT CTC TGT GGG CTG CGA-3′ [34]; *Clostridium perfringens (CPE gene)*, forward, 5′-TCC CCT TTC TAG ATA ACG ATT AAC AC-3′ and reverse, 5′-GTT AGC ATG CTG TTT TCT AAG TTA AAA CC-3′ [35]. Primers were synthesized by Sigma Genosys (Ishikari, Japan).

### Reverse transcription-polymerase chain reaction (RT-PCR)

To assess human CLDN4 mRNA expression, RT-PCR was performed with 0.5 μg total RNA extracted from HSC3 and HSC4 cells using an RNeasy kit (Qiagen, Germantown, MD, USA). The primer sets used were as follows; CLDN4, forward, 5′-CTC CAT GGG GCT ACA GGT AA-3′ and reverse, 5′-AGC AGC GAG TCG TAC ACC TT-3′ (NCBI reference sequence: NM_001305.4); CDH1, forward, 5′-TGC CCA GAA AAT GAA AAA GG-3′ and reverse, 5′-GTG TAT GTG GCA ATG CGT TC-3′ (Z13009.1); vimentin (VIM), forward, 5′-GAG AAC TTT GCC GTT GAA GC-3′ and reverse, 5′-TCC AGC AGC TTC CTG TAG GT-3′ (NM_003380.3); CD44, forward, 5′-CAT TCA AAT CCG GAA GTG CT-3′ and reverse, 5′-GTT GCC AAA CCA CTG TTC CT-3′ (FJ216964.1). Primers were synthesized by Sigma Genosys. The GAPDH mRNA was also amplified for use as an internal control (GenBank Accession No. NM_001101).

### Protein extraction

For preparing whole cell lysate, HSC3 and HSC4 cells were washed twice with cold PBS, harvested and lysed with 0.1% SDS-added RIPA-buffer (Thermo Fisher Scientific, Tokyo, Japan) [32]. Cell fractions were extracted by using a Cell Fractionation Kit (Abcam, Cambridge, MA, USA), according to the manufacturer’s instructions [36]. Protein assay was performed using a Protein Assay Rapid Kit (Wako Pure Chemical Corporation, Osaka, Japan).

### Immunoprecipitation

Immunoprecipitation was performed according to the method described previously [37]. Briefly, whole cell lysates were pre-cleaned in lysis buffer with protein A/G agarose (Santa Cruz) for 1 h at 4° C and subsequently centrifuged. The supernatants were incubated with antibodies against YAP1 (Abcam) or CLDN4 (4D3) or ZO-2 and protein A/G agarose for 3 h at 4° C. Precipitates were collected via centrifugation, washed five times with lysis buffer, solubilized with sample buffer (Sigma, 40 μg), and subjected to an immunoblot analysis with antibodies against CLDN4 (4D3), YAP1 (Abcam) or ZO-2 (Abcam).

### Cell surface labeling

Cell surface proteins were iodized with Na^131^I (Amersham, Aylesbury, UK) with iodination reagent (Pierce, Rockford, IL, USA) in HSC3 and HSC4 cells, which were added into the culture media and incubated with cells for 1 h. Then cells were washed with cold PBS for 3 times. Cells were subjected to protein extraction mentioned above.

### *In vitro* phosphorylation assay of recombinant proteins

Recombinant human YAP1 protein (Abcam, 1 μg) was mixed with recombinant human ZO-2 protein (Abcam, 1 μg) and/or recombinant human CLDN4 protein (Abcam, 1 μg) in a kinase buffer (20 mM Tris-HCl (pH 7.5), 5 mM MgCl_2_, 5 mM MnCl_2_, 1×phosphatase inhibitor, 1×protease inhibitor mixture) and incubated at 30° C for 30 min. Then recombinant human LATS1 protein (Abcam, 1 μg) and 20 μM ATP were added into the above given recombinant protein mixture and incubated at 30° C for 30 min. The reaction was stopped by adding 7 μl of 5× SDS sample dye, boiled at 100° C for 5 min, and subjected to SDS-PAGE.

### Statistical analysis

Statistical significance was calculated using chi-square, Fisher’s square test, and Kruskal-Wallis test with InStat software (GraphPad, Los Angeles, CA, USA). Statistical significance was defined as a two-sided *p*-value of < 0.05.

## SUPPLEMENTARY MATERIALS


