# Supplementary material for: Clostridium perfringens enterotoxin induces claudin-4 to activate YAP in oral squamous cell carcinomas
Source: Oncotarget. 2020 Jan 28;11(4):309–21. doi: 10.18632/oncotarget.27424 (PMC6996904; doi:10.18632/oncotarget.27424)
Supplement: Supplementary file 1 [file oncotarget-11-309-s001.pdf]

Clostridium perfringens enterotoxin induces claudin-4 to activate YAP in oral squamous cell carcinomas

SUPPLEMENTARY MATERIALS

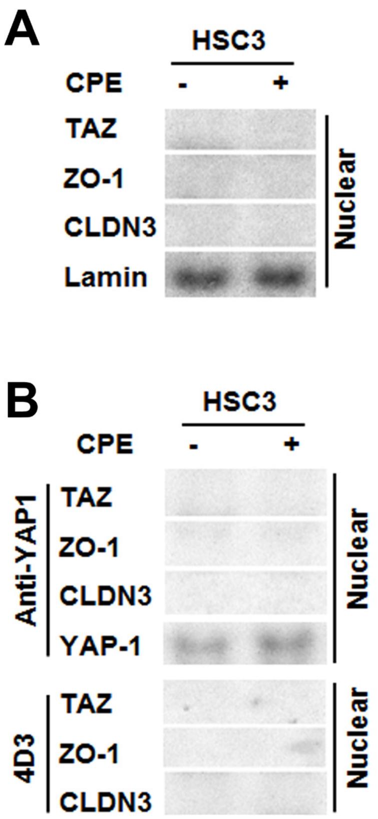

Supplementary Figure 1: Effect of CPE on interaction of CLDN3, TAZ and ZO-1 in OSCC cells. (**A**) Effect of CPE (10 µg/ml) on intranuclear protein levels of TAZ, ZO-1 and CLDN3 was examined by immunoblotting. Lamin was subjected as a loading control. (**B**) Inutanuclear interaction of TAZ, ZO-1 or CLDN3 with YAP1 or CLDN4 was examined by immunoprecipitation.
